# Recurrent aortic-arch pseudoaneurysm eroding into the left main bronchus following descending thoracic aortic stent repair

**DOI:** 10.1186/s13019-023-02112-9

**Published:** 2023-01-07

**Authors:** Lijun Duan, Xiaoqian Lu, Ruonan Pan, Dianbo Cao

**Affiliations:** Department of Radiology, The First Hospital of Jilin University, No. 71 of Xinmin Street, Changchun, Jilin 130021 China

**Keywords:** Behcet's disease, Vascular complications, Endovascular techniques, CT angiography

## Abstract

**Background:**

Behcet's disease is a form of systematic vasculitis that affects vessels of various sizes anywhere in the body. Aortic pseudoaneurysm is the most hazardous lesion in Behcet's disease and is associated with high mortality rate once rupture.

**Case presentation:**

In this report, we presented a patient with a known history of Behcet's disease, whose recurrent aortic-arch pseudoaneurysm eroding into the left main bronchus was identified after a 4-year duration of thoracic endovascular aortic repair for thoracic descending aortic pseudoaneurysm ruptured into the left lung. Repeated thoracic endovascular aortic repair combined with the chimney stent effectively controlled massive hemoptysis under the life-threatening circumstance, and the patient was in good condition at the 7-year follow-up.

**Conclusions:**

Thoracic endovascular aortic repair can be used as an effective and problem-solving treatment approach for thoracic aortic aneurysms eroded into the lung, even recurrent pseudoaneurysm after thoracic endovascular aortic repair in BD patients. Among the imaging methods assessing the technical success, outcome and complications, computed tomography angiography offers a fast, accessible and sensitive imaging modality.

## Background

Behcet’s disease (BD), while originally described as a triad of oral and genital ulcerations and relapsing ocular inflammation, has more to it than just that [1]. BD could affect various types of vessels manifesting as venous thrombosis and arterial pseudoaneurysm formation. Aortic involvement is one of the most severe manifestations of BD, and aortic aneurysm rupture has become the principal cause of death in BD with vascular involvement [2, 3]. The abdominal aorta is the most common site of pseudoaneurysm formation, while the thoracic aorta is a relatively rare location. Aortic pseudoaneurysm may be found as the first symptom or comes after previous treatment. Massive life-threatening hemoptysis occasionally occurs only when thoracic aortic pseudoaneurysm ruptures into the lung parenchyma or tracheobronchial trees. Traditional open repair of aortic aneurysm in BD patients is associated with a high recurrence rate of pseudoaneurysms at anastomotic sites [4]. Thoracic endovascular aneurysm repair (TEVAR) provides a favorable alternative for BD patients, as it refrains from the need for surgical aortic anastomosis. Even so, recurrent pseudoaneurysm is still a serious problem in BD patients [5]. Among the imaging methods assessing the technical success, outcome, and complications, noninvasive computed tomography angiography (CTA) offers a fast, accessible and sensitive imaging modality. We here reported such a patient with BD who developed recurrent pseudoaneurysm eroded into the left main bronchus after TEVAR for thoracic descending aortic pseudoaneurysm ruptured into the left pulmonary parenchyma. Repeated TEVAR combined with the chimney stent resulted in an effective outcome in controlling massive hemoptysis from aortic-arch pseudoaneurysms under the life-threatening condition.

## Case presentation

**Fig. 1:**
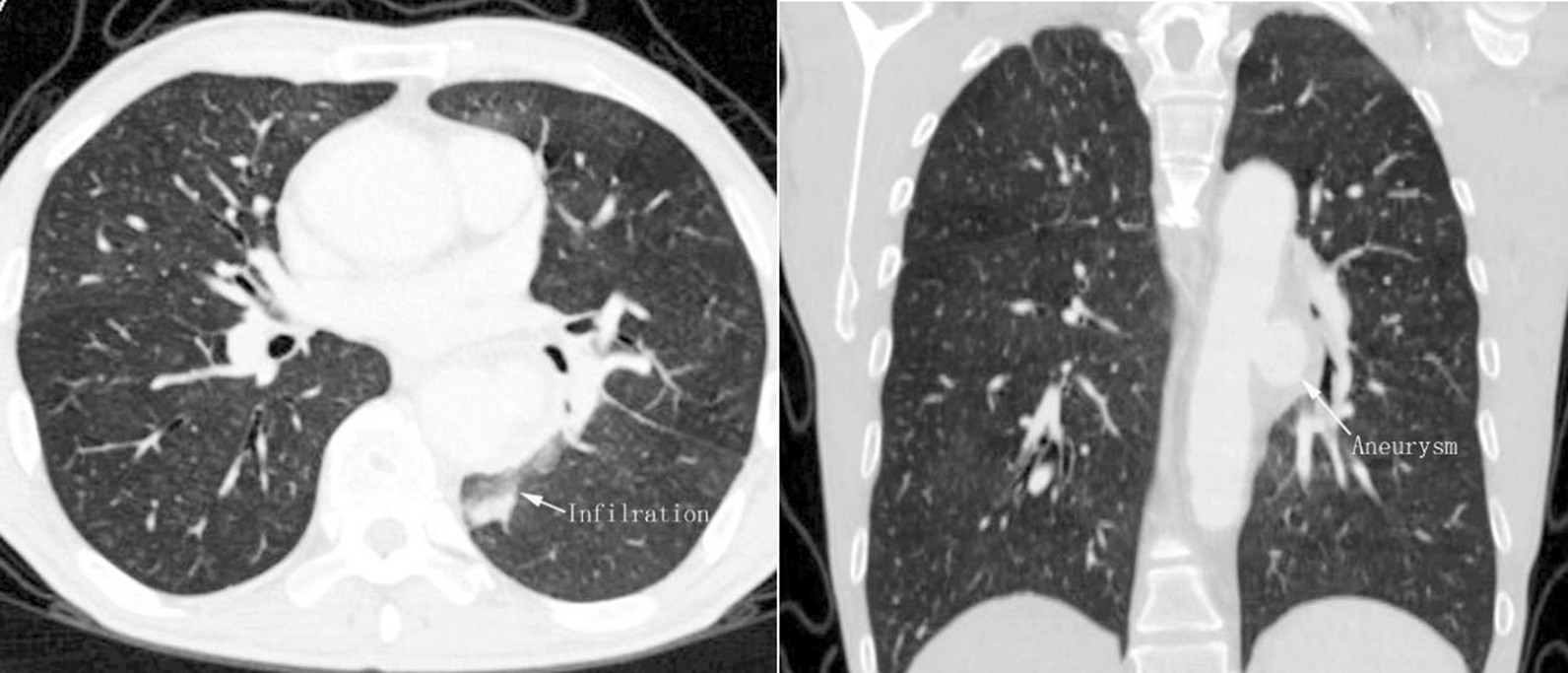
(Left), (right): Axially and coronally reconstructed CT images revealed a 21 × 30 mm saccular aneurysm with rupture into the superior segment of left lung lower lobe

A 47-year-old man with a confirmed 10-year diagnosis of BD was admitted to our hospital due to hoarse voice, recurrent hemoptysis, and dyspnea on exercise for about 2 weeks. The patient was diagnosed to have a thoracic aortic saccular aneurysm ruptured into the left lung on CTA 4 years previously (Fig. 1) and was treated by TEVAR. He recovered uneventfully and hemoptysis disappeared since then.

**Fig. 2:**
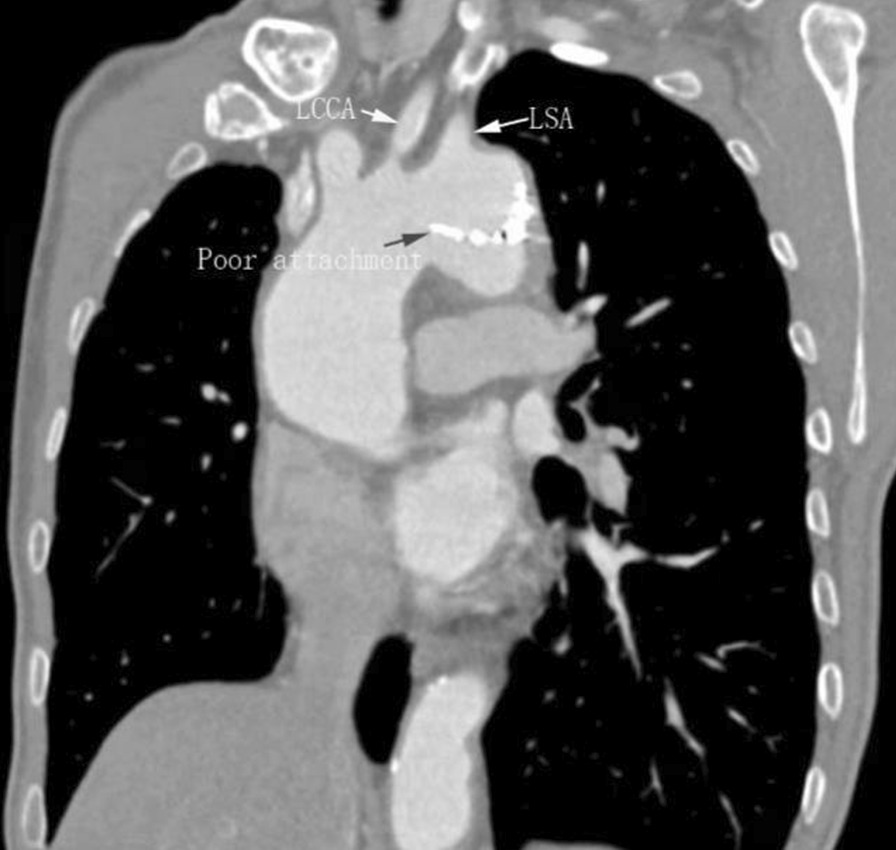
Obliquely reconstructed CTA one year later demonstrated that the proximal stent was poorly attached to the concave wall of aortic arch. LSA: left subclavian artery, LCCA: left common carotid artery

Approximately 12 months after initial TEVAR, follow-up CTA indicated that the stent was well positioned and poorly attached to the concave aspect of the aortic arch at the proximal part (Fig. 2), suggesting the risk of the future aneurysm.

**Fig. 3:**
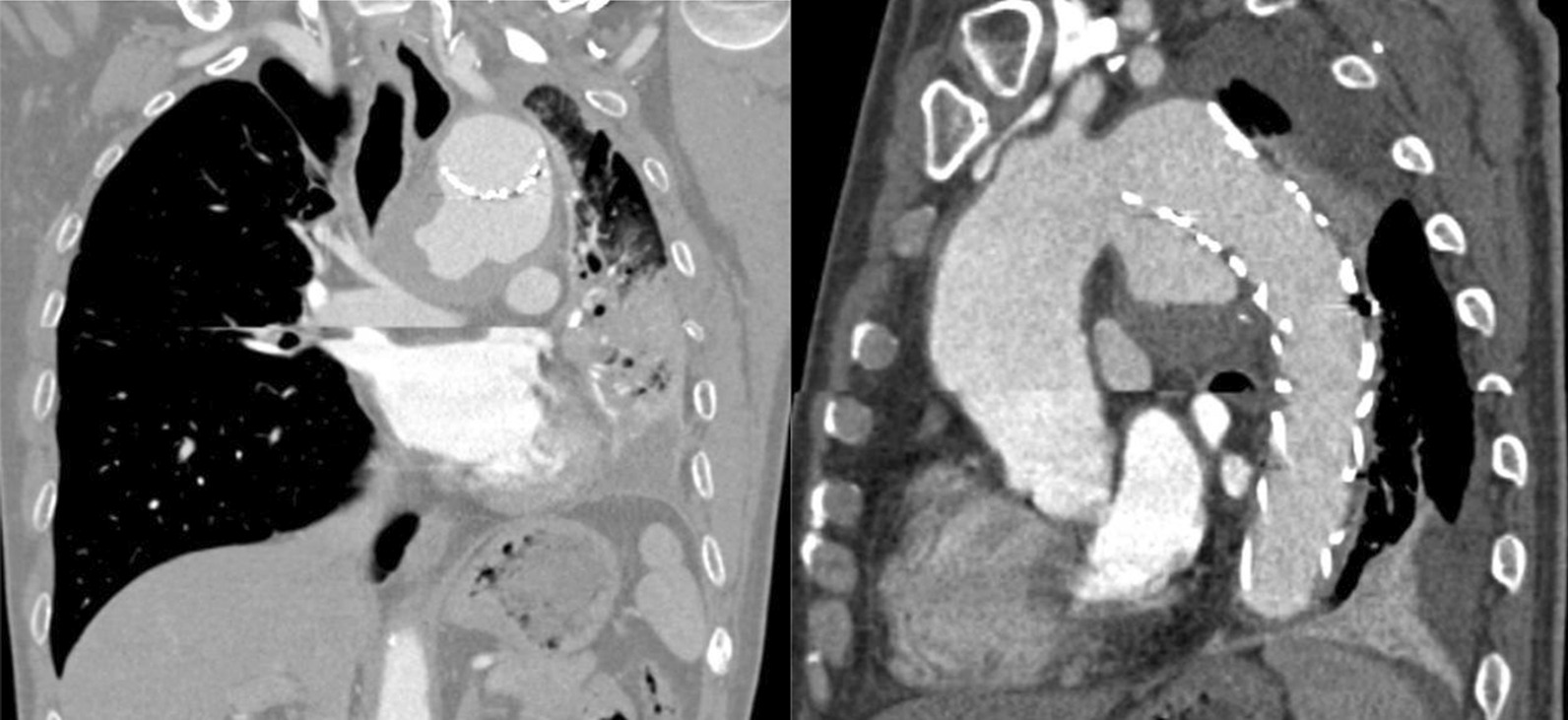
(Left): Coronal reconstruction image showed serious stenosis of the left main brochus and partial atelectasis of left lung due to aortic-arch pseudoaneurysm eroding into the main bronchus. (right): Sagittal reconstruction image demonstrated the mouth of pseudoaneurysm opposite to the orifice of the left common carotid artery

During this physical examination, his blood pressure was 122/69 mmHg, and his heart rate was 76 beats per minute. Decreased breath sounds were heard in the left lower-middle lung zone. The hemoglobin level was 109 g/L (reference range 130–175 g/L), C-reactive protein was 39.2 mg/L (reference range 0–3 mg/L), and erythrocyte sedimentation rate was 31 mm/1 h (reference range 0–15 mm/1 h). Chest CTA scan showed partial left lung atelectasis and serious stenosis of the left main bronchus invaded by aortic-arch pseudoaneurysm (Fig. 3) with a significant increase in size compared with the previous follow-up CT image.

**Fig. 4:**
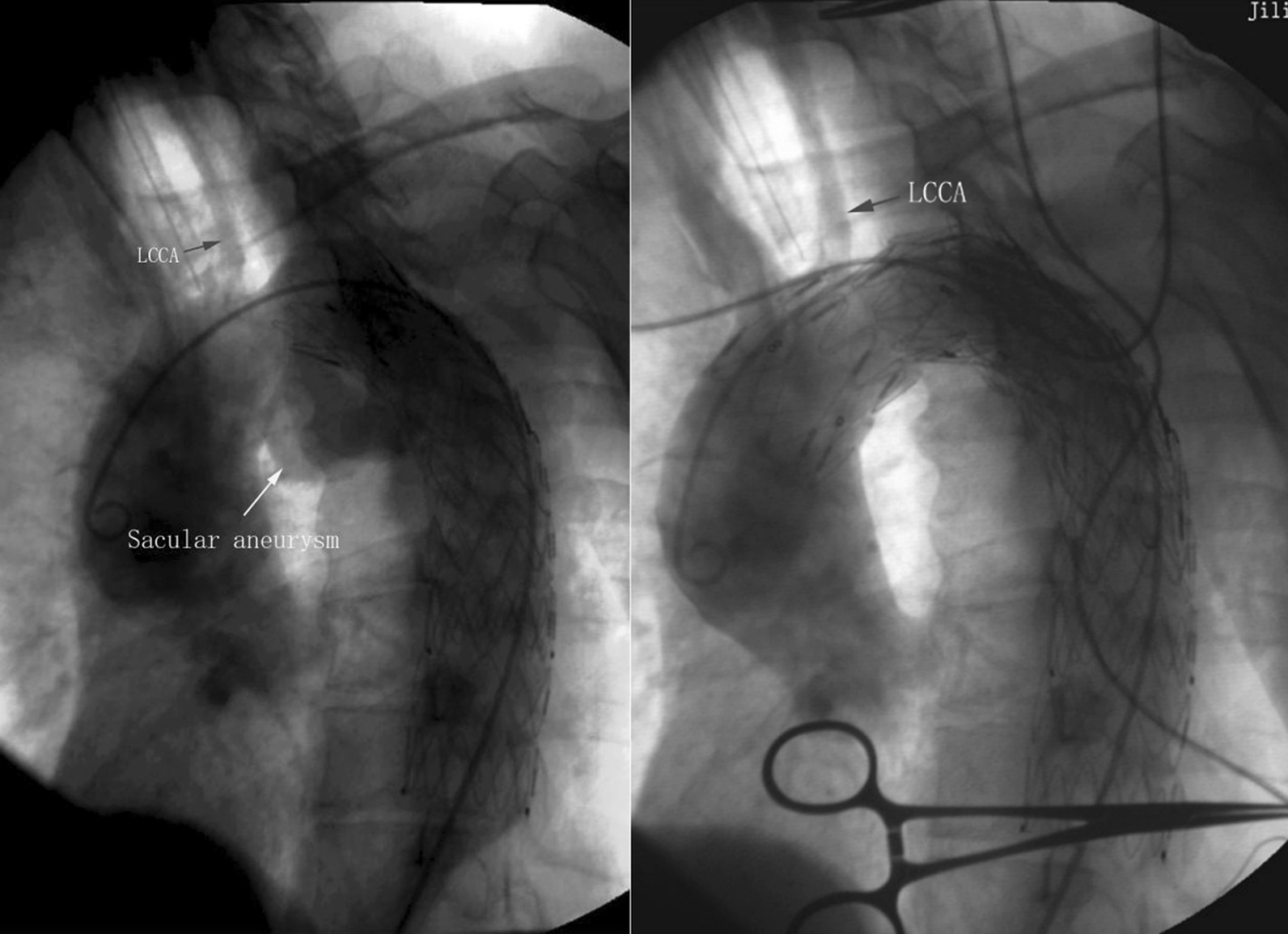
(Left): Intraoperative angiogram show saccular aneurysms located at the concave side of aortic arch just opposite to the orifice of left common carotid artery. LCCA: left common carotid artery. (right): A completion angiogram showed total occlusion of aortic-arch pseudoaneurysm

After multidisciplinary consultation, the patient was subjected to emergent TEVAR based on unexpected massive hemoptysis from new pseudoaneurysm at the proximal part of previous endovascular stent and patient’s poor condition. Under general anesthesia, the patient's right femoral artery was dissected and controlled. An angiographic catheter was introduced into the proximal end of the ascending aorta via right femoral artery puncture. Subsequent angiogram showed that pseudoaneurysm was just opposite to the orifice level of the left subclavian artery and left common carotid artery (Fig. 4). The inner diameter of the normal arota was 33 mm at the proximal portion of this pseudoaneurysm, so an endovascular stent graft (160 mm × 36 mm; XianJian, China) was deployed covering the poor attachment of the previous stent. Simultaneously, the orifice of the left common carotid artery and left subclavian artery were also occluded. Then, a self-expanding stent (60 mm × 10 mm; Gore Viabahn, America) with the chimney technique was retrogradely placed at the ostium of the left common carotid artery to preserve its perfusion through the transcarotid approach. The left subclavian artery was sacrificed because bilateral vertebral arteries equally supplied the brain in this patient. Repeat angiography showed complete occlusion of the pseudoaneurysm (Fig. 4) and the operational procedure was terminated.

**Fig. 5:**
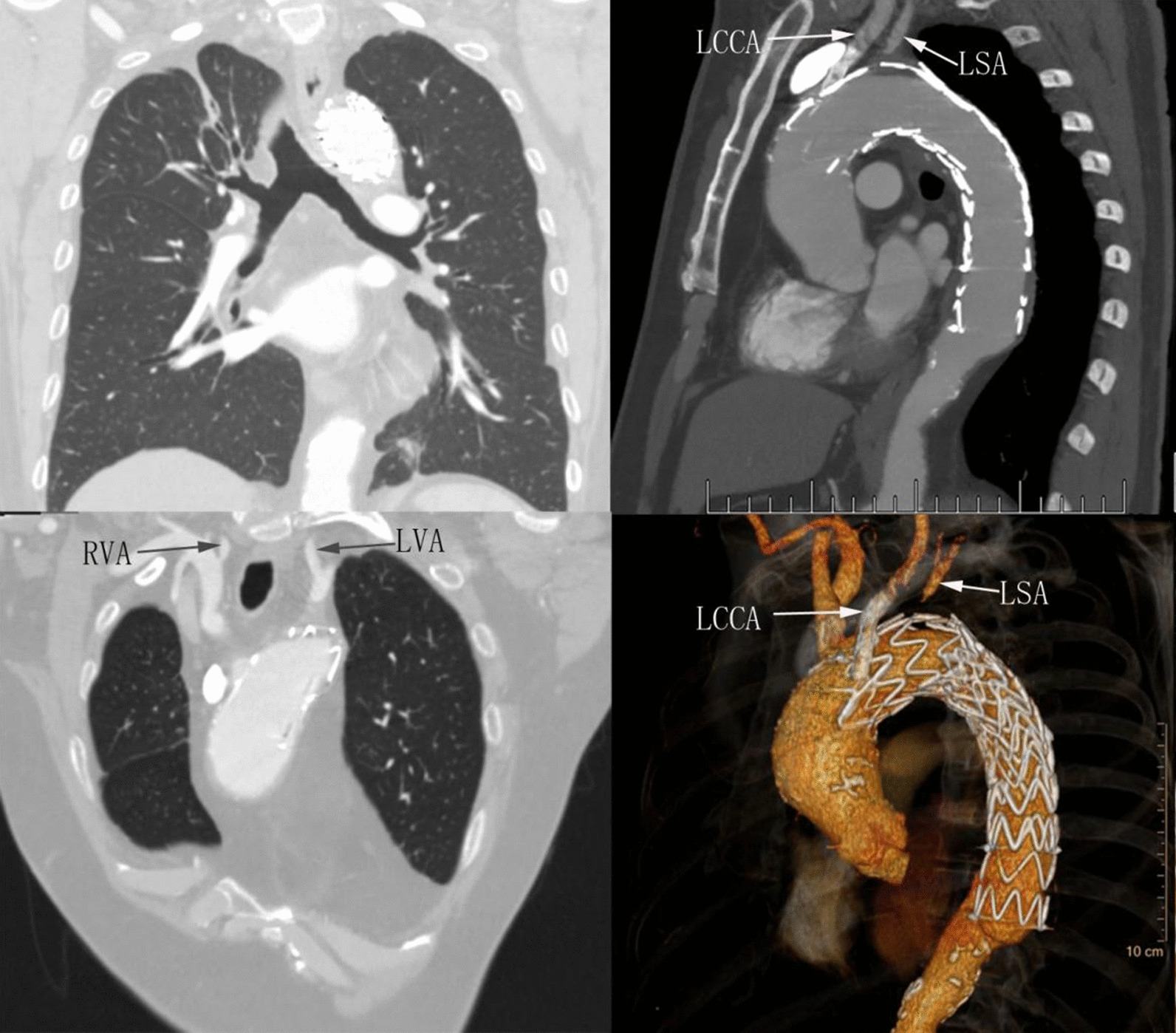
Postoperative CTA confirmed complete regression of the aortic-arch pseudoaneurysm, no bronchial stenosis, the patency of chimney stent and left subclavian artery. RVA: right vertebral artery, LVA: left vertebral artery, LSA: left subclavian artery, LCCA: left common carotid artery

On the tenth day of postoperative management, carotid color doppler showed left subclavian steal syndrome from retrograde blood flow of the left vertebral artery indicative of good collateral circulation. No change in the patient's neurologic status was present during the perioperative period and he was discharged 14 days later in a stable condition. The patient had been taking steroid hormone and immunosuppressive agents regularly since the second discharge. Chest CTA follow-up showed a satisfactory outcome at the interval of 84 months (Fig. 5), and no any complication related to TEVAR was detected again.

## Discussion and conclusions

**Fig. 6:**
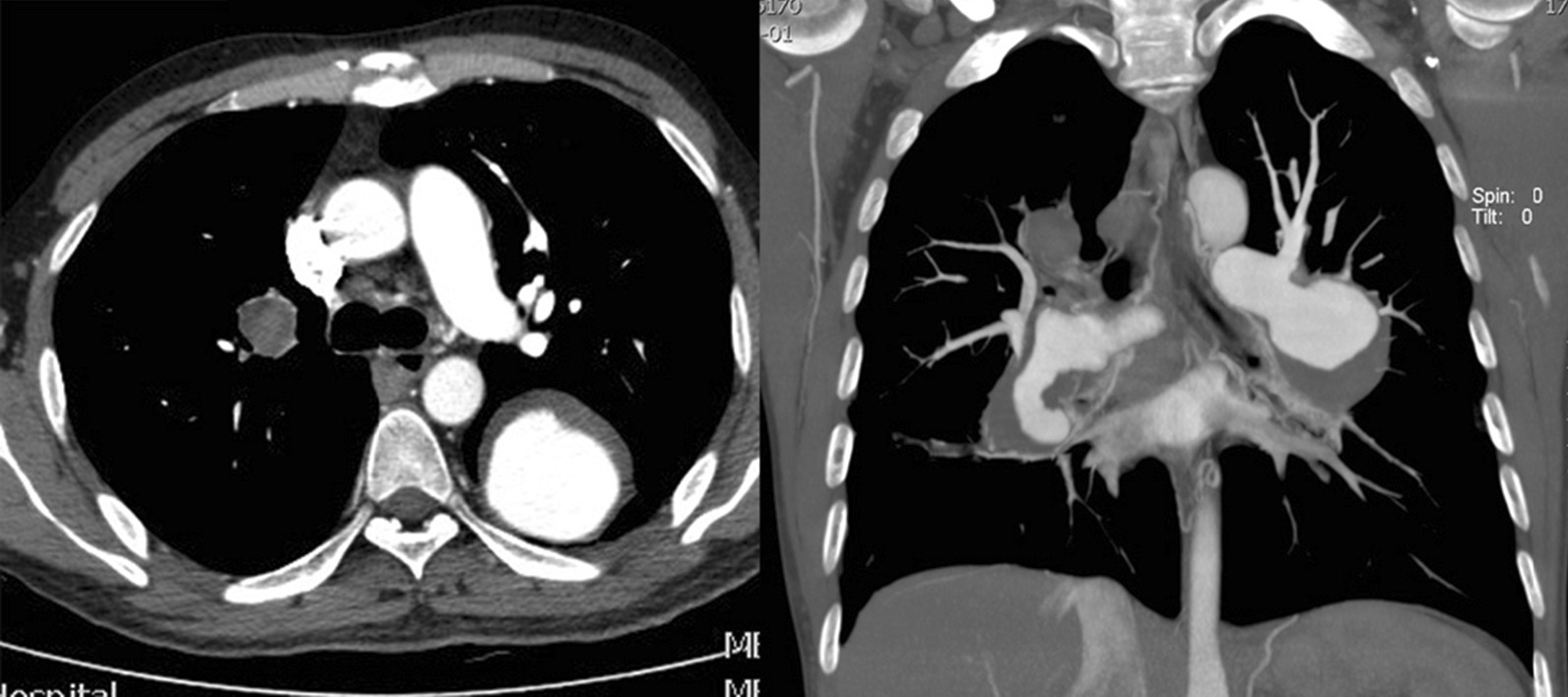
Multiple pulmonary aneurysms were detected in a 40-year-old man with a known diagnosis of BD in our unit

Behcet's disease (BD) is a rare, chronic, autoimmune, multisystem disorder that can cause inflammation of blood vessels anywhere in the body. BD could affect various types of vessels and cause variable kinds of pathologies. As arterial complications, the development of true or false aneurysms in great vessels accounts for 1–7% of BD patients [6]. The common sites of aneurysm related to BD have been reported as abdominal aortic aneurysm, pulmonary aneurysm (Fig. 6), carotid artery aneurysm, and lower extremity aneurysm. However, aortic pseudoaneurysm is the most disaster in BD, and aortic aneurysm rupture has become the principal cause of death in BD with vascular complications.

Aneurysm formation of the thoracic aorta is a rare but serious manifestation of BD. If thoracic aortic aneurysms (TAA) ruptures into the lung parenchyma or tracheobronchial tree, patients would suffer massive life-threatening hemoptysis, different from those common causes such as bronchiectasis, tuberculosis, fungal infections, and cancer. There was not any other evidence except for a definite history of BD in our patient when TAA was first identified to be responsible for initial hemoptysis, which suggested that the vascular lesion was not an isolated event but was associated with BD. Practically, detailed medical history and careful physical examination are of great value in occult onset patients. More interestingly, recurrent hemoptysis occurred 4 years later due to aortic-arch pseudoaneurysm invading into the left main bronchus based on CT evaluation. According to our speculation, the fistula developed between the aorta and the tracheobronchial tree possibly because erosion from the continuous pulsatile pressure from arterial blood flow and gradual enlargement of the pseudoaneurysm resulted in compression necrosis of the main bronchus. Radiographic screen like CTA provides direct evidence for the two episodes of hemoptysis in this patient.

The major problem of open surgical treatment for BD-associated aortic pseudoaneurysm is its tendency to develop recurrent false aneurysm at the anastomosis site, which occurs in 30–50% of patients [3]. TEVAR, introduced in the mid-1990s, provides a less invasive approach to treating acute and chronic thoracic aortic pathology with reduced surgery time and a more rapid recovery. Additionally, this therapy also causes less blood loss and has lower mortality rates. Considering these advantages, recent studies support endovascular repair and believe that it challenges conventional open surgical repair for the management of TAA, with systemic immunosuppressive and anti-inflammatory treatment before and after surgery in active BD [7–11]. Even so, recurrent pseudoaneurysm is still a serious problem in BD patients [12]. In our patient, thoracic descending pseudoaneurysm was candidate for endovascular repair based on the adequate anchor zone at his initial consultation. His recurrent aortic-arch pseudoaneurysm was highly suspected to be associated with poor attachment of the proximal stent based on the imaging appearances of a 1-year follow-up CTA. Meanwhile, vessel wall injuries or mechanical stress at the edge of the stent graft may trigger tissue inflammation together with arterial pulsation. Recurrent pseudoaneurysm at the margin of the stent graft requires timely intervention with additional stent graft.

With the continuous improvements in stent grafts and technical experiences, indications of TEVAR for aortic pathology are constantly enlarged. For those vascular lesions involving the aortic arch, important arterial branches, and a short anchor zone, the use of a common configuration of stent graft often leads to various types of severe complications. Parallel to the main aortic stent graft, the chimney technique uses a covered or bare stent to maintain blood flow to the vital organs, which could be indication to treat lesions with inadequate anchor zones. In our patient, the chimney stent was implanted into the left common carotid artery, while the left subclavian artery was sacrificed. A reversal of the left vertebral flow to the left subclavian artery was observed by the postoperative carotid Doppler, indicating the occlusion of its proximal end and the establish of collateral circulation. TEVAR combination with the chimney stent is a valid solution to the critical location of an aortic aneurysm in an emergency setting. A study combining TEVAR with the chimney technique revealed that the chimney-graft technique for aortic-arch pathologies is technically applicable in both elective and emergency situations, and is associated with satisfactory perioperative outcomes with a success rate of 90.2% [13]. Our successful management demonstrates that this approach may be an attractive therapeutic alternative to treat aortic-arch pseudoaneurysm for those high-risk surgical candidates. Meanwhile, the placement of the endovascular stent should be such that the proximal end does not lie in the convex of the proximal thoracic descending aorta, and it should be either positioned more proximally into the arch or distally into the straight portion of the thoracic aorta, as the lesson learned from our patient. The precise evaluation for therapeutic effect is easily obtained by CTA [14], similar to our screen.

In conclusion, TAA related to BD is rarely encountered, which is one of the rarest causes of life-threatening hemoptysis. TEVAR can be used as an effective and problem-solving treatment approach for TAA eroding into the lung, even recurrent pseudoaneurysm after initial TEVAR in BD patient. TEVAR combined with the chimney technique widens the indication for aortic pathology. Among the imaging methods assessing the technical success, outcome, and complications, CTA offers a fast, accessible, and sensitive imaging modality.

## Data Availability

We declared that materials described in the manuscript, including all relevant raw data, will be freely available to any scientist wishing to use them for non-commercial purposes, without breaching participant confidentiality.

## Abbreviations

BD: Behcet's disease
TEVAR: Thoracic endovascular aortic repair
TAA: Thoracic aortic aneurysms
CTA: Computed tomography angiography
LCCA: Left common carotid artery
LSA: Left subclavian artery
RVA: Right vertebral artery
LVA: Left vertebral artery

## References

[1] Fok M, Bashir M, Goodson N (2017). Thoracic aortic aneurysms in Behçet's disease. Rheumatology (Oxford).

[2] Marone EM, Diaco DA, Brioschi C (2018). Emergent endovascular treatment of thoracoabdominal aortic rupture in Behcet's disease. Ann Vasc Surg.

[3] Kural-Seyahi E, Fresko I, Seyahi N (2003). The long-term mortality and morbidity of Behçet syndrome: a 2-decade outcome survey of 387 patients followed at a dedicated center. Medicine (Baltimore).

[4] Hosaka A, Miyata T, Hoshina K (2014). Prognosis of arterial aneurysm after surgery in patients with Behçet's disease. Int Angiol.

[5] Honda S, Hirano F, Mouri M (2018). Aneurysm formation after stent grafting in vascular Behçet's disease. Arthritis Rheumatol.

[6] Gürler A, Boyvat A, Türsen U (1997). Clinical manifestations of Behçet’s disease: an analysis of 2147 patients. Yonsei Med J.

[7] Shen C, Li W, Zhang Y (2016). Outcomes of surgery for patients with Behcet's disease causing aortic pseudoaneurysm: a shift from open surgery to endovascular repair. Clinics (Sao Paulo).

[8] Balcioglu O, Ertugay S, Bozkaya H (2015). Endovascular repair and adjunctive immunosuppressive therapy of aortic involvement in Behçet's disease. Eur J Vasc Endovasc Surg.

[9] Park JH, Chung JW, Joh JH (2001). Aortic and arterial aneurysms in Behçet’s disease: management with stent-grafts–initial experience. Radiology.

[10] Kärkkäinen JM, Pather K, Tenorio ER (2019). Should endovascular approach be considered as the first option for thoraco-abdominal aortic aneurysms?. J Cardiovasc Surg (Torino).

[11] Tenorio ER, Dias-Neto MF, Lima GBB (2021). Endovascular repair for thoracoabdominal aortic aneurysms: current status and future challenges. Ann Cardiothorac Surg.

[12] Zhang SH, Zhang FX (2017). Behcet's disease with recurrent thoracic aortic aneurysm combined with femoral artery aneurysm: a case report and literature review. J Cardiothorac Surg.

[13] Yang J, Xiong J, Liu X (2012). Endovascular chimney technique of aortic arch pathologies: a systematic review. Ann Vasc Surg.

[14] Qi L, Cai J, Mao D (2019). Use of contrast-enhanced computed tomographic imaging to diagnose and evaluate Behçet's disease with vascular complications. Exp Ther Med.

